# Brain *CHID1* Expression Correlates with *NRGN* and *CALB1* in Healthy Subjects and AD Patients

**DOI:** 10.3390/cells10040882

**Published:** 2021-04-13

**Authors:** Paola Castrogiovanni, Cristina Sanfilippo, Rosa Imbesi, Grazia Maugeri, Debora Lo Furno, Daniele Tibullo, Alessandro Castorina, Giuseppe Musumeci, Michelino Di Rosa

**Affiliations:** 1 Department of Biomedical and Biotechnological Sciences, Human Anatomy and Histology Section, School of Medicine, University of Catania, 95123 Catania, Italy; pacastro@unict.it (P.C.); roimbesi@unict.it (R.I.); grazia.maugeri@libero.it (G.M.); gmusumeci@unict.it (G.M.); 2 IRCCS Centro Neurolesi Bonino Pulejo, Strada Statale 113, C.da Casazza, 98124 Messina, Italy; cristina.sanfilippo@irccsme.it; 3 Department of Biomedical and Biotechnological Sciences, Physiology Section, University of Catania, 95123 Catania, Italy; lofurno@unict.it; 4 Department of Drug Science, Biochemistry Section, University of Catania, 95125 Catania, Italy; d.tibullo@unict.it; 5 Laboratory of Cellular and Molecular Neuroscience (LCMN), School of Life Sciences, Faculty of Science, University of Technology Sydney, Broadway, NSW 2007, Australia; alessandro.Castorina@UTS.edu.au; 6 Laboratory of Neural Structure and Function (LNSF), School of Medical Sciences (Anatomy & Histology), Faculty of Medicine and Health, University of Sydney, Sydney, NSW 2006, Australia

**Keywords:** *CHID1*, Alzheimer’s disease, chitinases, bioinformatics

## Abstract

Alzheimer’s disease is a progressive, devastating, and irreversible brain disorder that, day by day, destroys memory skills and social behavior. Despite this, the number of known genes suitable for discriminating between AD patients is insufficient. Among the genes potentially involved in the development of AD, there are the chitinase-like proteins (CLPs) CHI3L1, CHI3L2, and CHID1. The genes of the first two have been extensively investigated while, on the contrary, little information is available on CHID1. In this manuscript, we conducted transcriptome meta-analysis on an extensive sample of brains of healthy control subjects (n = 1849) (NDHC) and brains of AD patients (n = 1170) in order to demonstrate CHID1 involvement. Our analysis revealed an inverse correlation between the brain *CHID1* expression levels and the age of NDHC subjects. Significant differences were highlighted comparing CHID1 expression of NDHC subjects and AD patients. Exclusive in AD patients, the *CHID1* expression levels were correlated positively to calcium-binding adapter molecule 1 (*IBA1*) levels. Furthermore, both in NDHC and in AD patient’s brains, the *CHID1* expression levels were directly correlated with calbindin 1 (*CALB1*) and neurogranin (*NRGN*). According to brain regions, correlation differences were shown between the expression levels of *CHID1* in prefrontal, frontal, occipital, cerebellum, temporal, and limbic system. Sex-related differences were only highlighted in NDHC. CHID1 represents a new chitinase potentially involved in the principal processes underlying Alzheimer’s disease.

## 1. Introduction

Neurodegeneration indicates a progressive structural, functional, and molecular alteration of neurons, with consequent progressive cell degeneration. As regards, neurodegenerative diseases, such as amyotrophic lateral sclerosis, Alzheimer’s disease (AD), Parkinson’s disease, and Huntington’s disease, they are characterized by neurodegenerative phenomena [1]. The molecular mechanisms that regulate these diseases are also present in aging and neurodegenerative diseases. The damage to neurons revealed during aging can be considered to be exacerbated in neurodegenerative diseases [2]. The neurodegenerative processes are triggered by genetic predisposition factors linked to intrinsic susceptibility and epigenetic mechanisms associated with environmental factors, with aging itself increasing the risk [2]. Being able to understand the principal molecules that regulate the cellular mechanisms of the neuroglia represents a new strategy to counteract the development and progression of neurodegenerative diseases. Among the main neurodegenerative diseases, Alzheimer’s disease (AD) represents one of the most common with still unknown pathogenesis [3]. Recently, several investigations have demonstrated the decisive role of both neuroinflammation and the toxicity carried out by amyloid-beta (Aβ) peptide on central nervous system (CNS) neurons [4]. Aβ peptides tend to precipitate forming microaggregates, commonly called “soluble Aβ oligomers”, protofibrils, and fibrils which tend to accumulate in the brains of AD patients, forming the more well-known insoluble “amyloid plaques” [5]. Consequently, the accumulation of Aβ peptides results in neuron death and local immune activation, which leads to synaptic and cognitive dysfunctions. Several proteins, such as chitinases [6], calcium-binding adapter molecule 1 (IBA1) [7], platelet endothelial cell adhesion molecule (CD31) [8], and calbindin 1 (CALB1) [9], could be considered new potential markers of the cellular architecture alteration of the nervous system parenchyma.

Mammalian chitinases consisting of chitinase acid (CHIA) [10], chitotriosidase (CHIT1) [11], chitinase 3-like protein 1 (CHI3L1) [12], chitinase 3-like protein 2 (CHI3L2) [13], and chitinase domain-containing protein 1 (*CHID1*) [14] exert important biological roles in different cell types, such as polarized macrophages [15], dendritic cells [16], osteoclasts [17], and several cells with high proliferative activity [18,19]. CHI3L1, CHI3L2, and *CHID1* are chitin-binding proteins (CLPs) that lack chitin-hydrolyzing activity but possess cytokine-like and growth factor-like properties. The expression of CLPs has been related to several pathological phenomena with an inflammatory etiogenesis [20]. In recent years, different roles have been attributed to CLPs, such as tissue remodeling during inflammation, differentiation, and maturation of macrophages. Its high levels have been associated with various pathological disorders such as diabetes [21], osteoarthritis [22], and asthma [23]. Very little is known about the role of *CHID1*, which is also known as stabilin-1-interacting chitinase-like protein (SI-CLP). It has been shown that interact with the protein STAB1. The protein structure presents carbohydrate binding sites, which could be involved in carbohydrate catabolysis. *CHID1* is a marker for alternative macrophage activation. *CHID1* was abundantly detected in bronchoalveolar lavage from patients with chronic inflammatory disorders of the respiratory tract and human peripheral blood leukocytes [24]. *CHID1* secretion is mediated by its interaction with the endocytic/sorting receptor stabilin-1 and is activated by Th2 cytokines [24]. Our recent papers detected the existence of *CHID1* in pediatric brain tumors [14], macrophages infected with HIV-1 virus [25], and LOAD brain patients [13,26]. Although CHID1 was initially associated with alternative activated macrophages, evidence suggests that during neurodegenerative processes, its expression is abundant in the nucleoplasm of microglia [27]. Currently, there is no exhaustive information on its possible correlation with markers of neuroimmune activation, alteration of the blood–brain barrier (BBB), and neuronal transmission. Starting from this data, we selected IBA1, CD31, and CALB1, new neurodegeneration markers, in order to identify potential a correlation with *CHID1* in the CNS.

Allograft inflammatory factor 1 (AIF-1), also known as ionized calcium-binding adapter molecule 1 (IBA1), is mainly produced by the innate immune cells specifically in monocyte-derived macrophages [28], neutrophils [29], and microglia [30] in response to the cytokine IFN-γ [31]. It has been shown to be overexpressed during the activation of the immune cells in CNS injuries. As for its distinctive characteristics, it can be considered a glia marker of activation [32]. Recently, it has been hypothesized that IBA1 can regulate microgliosis by determining the scavenger of cellular debris, produced as waste during neurodegenerative processes, the recruitment of oligodendrocytes, and the reorganization of CNS cellular structure [33].

Platelet endothelial cell adhesion molecule (PECAM-1) is also known as cluster of differentiation 31 (CD31), highly expressed on neutrophils, monocytes, lymphocytes, platelets, and endothelial cells [34]. It was demonstrated that it plays a role in cell adhesion by mediating the diapedesis of the immune system cells through the modification of the vascular wall, suggesting a role potential as a vascular integrity marker [34]. Currently, it has been shown to have direct involvement in the regulation of BBB integrity [35]. Furthermore, it has also been shown that CD31 plays a relevant role in Aβ-related cerebral vascular disorders pathogenesis, such as AD [36], and is involved in the pathological molecular mechanism of neurological disease and in neuroHIV neuroinflammation [37].

Calbindin 1 (CALB1) is a calcium-binding and buffering protein, and it has been highlighted that it has a relevant role in preventing neuron death [38]. Alterations in the expression levels of this gene have been highlighted in patients with Huntington’s disease [39]. Due to its function, an increase in its expression has been associated with a protective role in various neurological diseases. Actually, the increase in CALB1 protein induces neurite outgrowth in dopaminergic neuronal cells and provides protection to dopaminergic neurons against pathological processes in Parkinson’s disease [40]. With aging, calbindin-containing neurons cells in the basal forebrain gradually die, and this process is exacerbated in AD patients [41]. During human immunodeficiency virus encephalitis (HIVE), neuronal damage could be produced by CALB1 reduction and increased neuron intracellular calcium [42].

Neurogranin (NRGN or Ng) is a 78-amino-acid-long post-synaptic protein, highly expressed in the brain, predominantly in dendritic spines of neurons in the amygdala, hippocampus, cerebral cortex, and other associative cortical areas [43,44]. Its protein actively participates in signal transduction in the protein kinase C signaling pathway. The main function is carried out at a postsynaptic level, regulating the availability of calmodulin, binding to it via an IQ motif (amino acid 33–46) in the absence of calcium. It has been shown that the mRNA and protein levels in the hippocampus decrease with age and are related to CNS dysfunction [45]. Its concentration in cerebrospinal fluid (CSF) is considered an index of synaptic dysfunction in neurodegeneration. High levels have been shown in the CSF of AD patients compared to healthy controls [46]. Data analysis from brain tissue indicates a decrease of *NRGN* concentrations in both the frontal cortex [47], parietal cortex [48], and hippocampus [47]. There is a direct link between NRGN and CHI3L1 so as to be able to consider the two proteins of the independent markers of synaptic degeneration and neuroinflammation in AD [49].

In this study, we investigated the expression levels of *CHID1* in NDHCS subjects and brain biopsies of AD patients, and the possible correlations with aging, in different brain regions. To do this, we collected and clustered the transcriptome of more than 3000 brain samples present in NCBI in the GEODataSet section. Furthermore, we hypothesized that the *CHID1* expression levels in the AD patient brain samples were correlated with markers of microglial activation (*IBA1*), vascular integrity (CD31), and neuronal death (CALB1) and neurogranin (*NRGN*), all phenomena characterizing the brain of AD patients.

## 2. Materials and Methods

### 2.1. Data Selection

For our analysis, we have collected 18 microarray datasets of brain data biopsies of non-demented subjects who died from causes not attributable to neurodegenerative diseases, and deceased patients suffering from AD. The transcriptome datasets were downloaded from NCBI Gene Expression Omnibus (GEO) database (http://www.ncbi.nlm.nih.gov/geo/, accessed on 9 January 2021) [50]. MeSH terms “Brain region”, “Human”, and “Alzheimer’s disease” were used to identify human potential datasets of interest. The selected datasets are shown in Table 1.

Harvard Brain Tissue Resource Center (HBTRC); Mount Sinai Medical Center Brain Bank (MSBB); National Institute on Aging (NIA); Stanley Medical Research Institute (SMRI); University of Maryland Brain Bank (UMBB); UK Brain Expression Consortium (UKBEC); Medical Research Council (MRC) London Neurodegenerative Diseases Brain Bank (from now on referred to as MRC-LBB); University of Pittsburgh’s Brain Tissue Donation Program (PBTDP); Neuropathology Consortium of the Stanley brain collection (Stanley Medical Research Institute, US) (SMRIC); Kyushu University (KU); BrainNet Europe network (BNEN); Mount Sinai/JJ Peters VA Medical Center Brain Bank (MSBB); Alzheimer’s Disease Centers (ADCs); Brain Bank of the Alzheimer’s Disease Research Center at the University of Kentucky (BBADRCUK); Brain and Tissue Bank for Developmental Disorders at the University of Maryland (BTBDDUM); NDHC = non-demented healthy controls subjects; AD = Alzheimer’s disease patients.

Each brain with AD was age-matched to a healthy brain to rule out any differences due to routine aging. Furthermore, we stratified the samples according to sex and age as shown in Table 2. Five groups were obtained: middle-age (53–65 years), senior (66–75 years), elderly (76–89 years), nonagenarian (90–99 years) and centenarian (>100 years) [12].

All brains sample analyzed were grouped into 8 main brain regions (prefrontal, frontal, occipital, cerebellum, temporal, cingulate, diencephalon, limbic system). Complete brain regions details examined are presented in Table 3.

### 2.2. Clinical and Neuropathological Criteria

A total of 1853 data points from frozen tissue samples were selected belonging to subjects who did not die from causes related to neurological diseases that we have identified as non-demented healthy controls subjects (NDHC) (70.63 ± 12.45 years) and 1170 samples taken from AD patients (82.03 ± 9.29 years). Most of the samples analyzed were obtained from public brain databases (Table 1). Postmortem interval (PMI), sample pH, and RNA integrity number (RIN) were elements of pre-selection by the authors of the reference microarray datasets and, subsequently, were bases for further exclusion in our analysis. For the diagnosis of AD, we took into consideration the investigations carried out by the authors of the individual datasets (Consortium to Establish a Registry for Alzheimer’s Disease (CERAD) guidelines, progressive decline in memory, cognitive deficits in two or more areas, MMSE , CDR, Braak stage, general and regional atrophy, gray and white matter atrophy, ventricular enlargement, and cataloged neuropathological diagnosis by pathologists based on e.g., neurofibrillary tangle (NFT) counts). As regards the cognitive integrity of the healthy subjects included in our analysis, we took into account the investigations and cognitive tests carried out by the authors of the microarray datasets listed in Table 1 (memory complaints, history of memory complaints, normal cognitive function documented by scoring age and education adjusted, mini-mental status examination (MMSE), and global clinical dementia rating, CDR).

### 2.3. Data Processing and Experimental Design

In order to process and identify significantly differentially expressed genes (SDEG) in all selected datasets, we used MultiExperiment Viewer (MeV) software (The Institute for Genomic Research (TIGR), J. Craig Venter Institute, USA). In cases where multiple genes probes insisted on the same GeneID, we used those with the highest variance. The significance threshold level for all datasets was *p* < 0.05. Statistically significant genes were selected for further analysis. For all datasets we performed a statistical analysis with GEO2R, applying a Benjamini and Hochberg FDR (false discovery rate) to adjust *p* values for multiple comparisons [69,70,71,72].

### 2.4. Statistical Analysis

For statistical analysis, Prism 8.0.2 software (GraphPad Software, San Diego, CA, USA) was used. Based on Shapiro–Wilk test, almost all data were normal, so parametric tests were used. Significant differences between groups were assessed using the ordinary one-way ANOVA test, and Tukey’s multiple comparisons test correction was performed to compare data between all groups. Correlations were determined using Pearson correlation. All tests were two-sided, and significance was determined at adjusted *p* value of 0.05. All MD selected were transformed for the analysis in Z-score intensity signal. Z score is constructed by taking the ratio of weighted mean difference and combined standard deviation according to Box and Tiao (1992) [73]. The application of a classical method of data normalization, z-score transformation, provides a way of standardizing data across a wide range of experiments and allows the comparison of microarray data independent of the original hybridization intensities. The z-score is considered a reliable procedure for this type of analysis and can be considered a state-of-the-art method, as demonstrated by the numerous articles [74,75,76,77,78,79,80,81,82,83,84,85].

The efficiency of each biomarker was assessed by the receiver operating characteristic (ROC) curve analyses [86,87]. Nonparametric ROC curves analyzed AD vs. NDHC. The area under the ROC curve (AUC) and its 95% confidence interval (95% CI) indicate diagnostic efficiency. The accuracy of the test with the percent error is reported [88].

## 3. Results

### 3.1. Sex-Dependent Differences in CHID1 Brain Expression of NDHC Subjects and AD Patients 

For our analysis, we have collected 1170 AD patients and 1849 non-demented healthy control subjects (NDHC) in order to verify the *CHID1* expression levels. The samples were stratified into five groups according to age (middle-age, senior, elderly, nonagenarian, and centenarian). A summary of sample sizes included in this study is described in Table 2.

**Figure 1:**
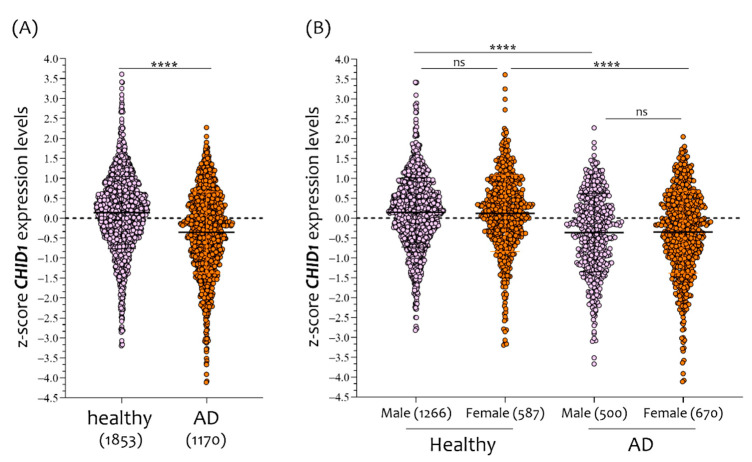
Sex-dependent difference in *CHID1* brain expression of NDHC subjects. Analysis of *CHID1* expression levels in 1853 NDHC brains subjects and 1170 AD brains patients (**A**), placed in order according to the sex (**B**). Data in the figure are indicated as z-score intensity expression levels and presented graphically as violin dot plots. *p* values < 0.05 were considered to be statistically significant (ns = not significant; **** *p* < 0.00001).

Brain *CHID1* RNA expression levels were significantly lower in patients with AD as compared to NDHC subjects (Figure 1A,B). When comparing the whole-brain *CHID1* expression levels of NDHC and AD according to the sex, we found no significant difference (Figure 1B). 

**Figure 2:**
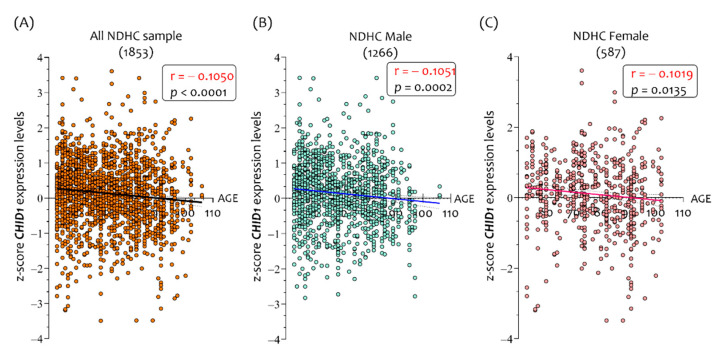
Correlation analysis between *CHID1* brain expression levels and the age of NDHC subjects. Correlation analysis between *CHID1* and the age of NDHC subjects (**A**), sorted according to the sex in brain samples of 1266 males (**B**) and 587 females (**C**). Data in the figure are indicated as z-score intensity expression levels and presented graphically as dot plots. *p* values < 0.05 were considered to be statistically significant.

There was a moderate inverse correlation between *CHID1* brain expression levels in NDHC and age (r = −0.1050, *p* < 0.0001) (Figure 2A). A significant inverse correlation in *CHID1* brain expression levels has been observed in both males (r = −1051, *p* = 0.0002) (Figure 2B) and females (r = −1019, *p* = 0.0135) (Figure 2C) of NDHC subjects. 

**Figure 3:**
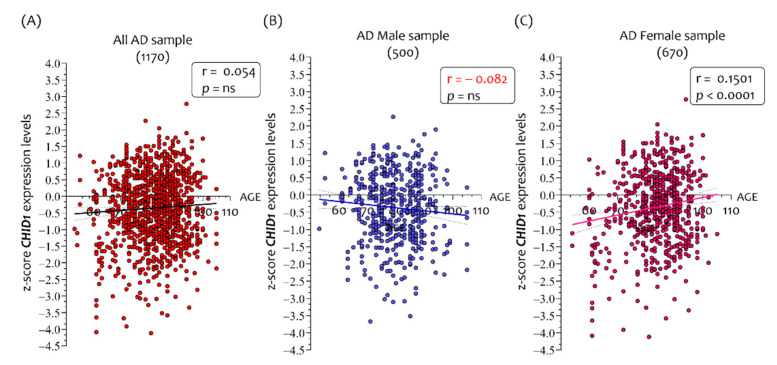
Correlation analysis between *CHID1* brain expression levels and the age of AD patients from which the samples were taken. Correlation analysis between *CHID1* and the age of AD patients (**A**), sorted according to the sex in brains of 1266 males (**B**) and 587 females (**C**). Data in the figure are indicated as z-score intensity expression levels and presented graphically as dot plots. *p* values < 0.05 were considered to be statistically significant.

The correlation analysis between *CHID1* expression levels and age in the brains of AD patients showed conflicting results (Figure 3). Brain expression of *CHID1* did not correlate with age in AD patients (r = 0.050, *p* = ns) (Figure 3A). In AD male patients, we observed the same trend for all samples (r = 0.082, *p* = ns) (Figure 3B), while in females, a positive correlation with age was highlighted (r = 0.1501, *p* < 0.00001) (Figure 3C).

### 3.2. Neurodegeneration Markers Correlated with CHID1 Expression Levels in the CNS

In dividing the samples of the 1170 AD patients according to sex, 500 were males and 670 were females, and of the 1849 NDHC subjects, 1264 were males and 585 were females (Table 2). We sorted the samples according to gender and carried out a comparative analysis of expression levels.

**Figure 4:**
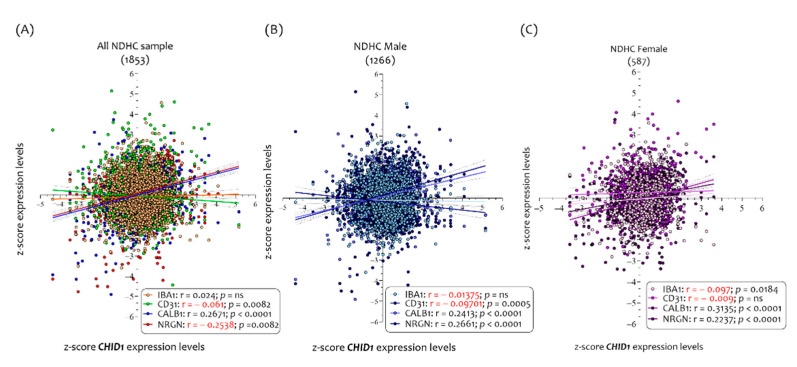
Correlation analysis between brain *CHID1* expression levels and neurological markers in NDHC subjects. Analysis of the correlation between the *CHID1*, *IBA1*, CD31, *CALB1*, and *NRGN* expression levels in all brains (1853) (**A**), and in brains sorted according to 1266 males (**B**) and 587 females (**C**) belonging to NDHC subjects. Data in the figure are indicated as z-score intensity expression levels and presented graphically as dot plots. *p* values < 0.05 were considered to be statistically significant.

The analysis of 1849 NDHC brains show that the *CHID1* expression levels were correlated with CD31 (r = −0.061, *p* = 0.0082), *CALB1* (r = 0.2671, *p* < 0.0001), and *NRGN* (r = −0.2538, *p* < 0.0001). No correlation was observed between *CHID1* and *IBA1* expression levels (Figure 4A). 

In the 1264 NDHC male brain samples, we showed that the *CHID1* expression levels were correlated with CD31 (r = −0.097, *p* = 0.0005), *CALB1* (r = 0.2413, *p* < 0.0001), and *NRGN* (r = 0.2661, *p* < 0.0001) (Figure 4B). In addition, for NDHC male brains, no correlation was observed between *CHID1* and *IBA1*.

When we analyzed the 585 NDHC female brain samples, we demonstrated that *CHID1* expression levels correlated both to *CALB1* (r = 0.3135, *p* < 0.0001) and to *NRGN* (r = 0.2237, *p* < 0.0001), but not to CD31 (r = −0.009, *p* = ns) (Figure 4B). Contrary to data highlighted in the brains of NDHC males, *CHID1* expression was negatively correlated with *IBA1* in females (r = −0.097, *p* = 0.0184) (Figure 4C).

**Figure 5:**
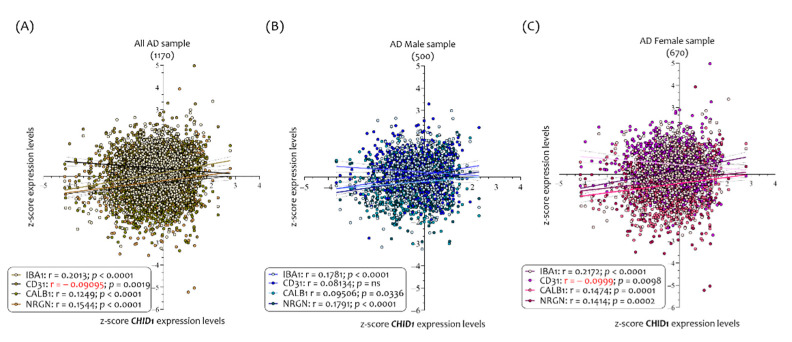
Correlation analysis between *CHID1* brain expression levels and neurological markers in AD patients. Analysis of correlation between the *CHID1*, *IBA1*, CD31, *CALB1*, and *NRGN* expression levels in all brains (1170) (**A**), and in brains sorted in 500 males (**B**) and 670 females (**C**), belonging to AD patients. Data in the figure are indicated as z-score intensity expression levels and presented graphically as dot plots. *p* values < 0.05 were considered to be statistically significant.

Partially overlapping results were highlighted in AD brains. The analysis of 1170 AD brains show that *CHID1* expression levels were correlated with *IBA1* (r = 0.2013, *p* < 0.0001), CD31 (r= −0.09, *p* = 0.0019), *CALB1* (r = 0.1249, *p* < 0.0001), and *NRGN* (r = 0.1544, *p* < 0.0001) (Figure 5A).

In analyzing the *CHID1* expression level in the brains of 500 AD males, we have been able to ascertain that its expression levels were correlated with *IBA1* (r = 0.1781, *p* < 0.0001), *CALB1* (r = 0.095, *p* =0.036), and *NRGN* (r = 0.1791, *p* < 0.00001) but not to CD31 (r = 0.081, *p* = ns) (Figure 5B).

Regarding the 670 AD female brains, the results show that the *CHID1* expression levels correlated with *IBA1* (r = 0.2172, *p* < 0.0001), CD31 (r = −0.09, *p* = 0.0098), *CALB1* (r= 0.1474, *p* = 0.0001), and *NRGN* (r = 0.1414, *p* = 0.0002) (Figure 5C).

### 3.3. CHID1 Levels Are Differentially Expressed in Eight Brain Regions 

We deepened our investigation by comparing the expression levels of *CHID1* in the different brain regions according to sex. Fifty-eight brain portions were grouped into 8 main brain regions (prefrontal, frontal, occipital, cerebellum, temporal, cingulate, diencephalon, and limbic system). Complete brain regions details examined in this paper are available in Table 3.

**Figure 6:**
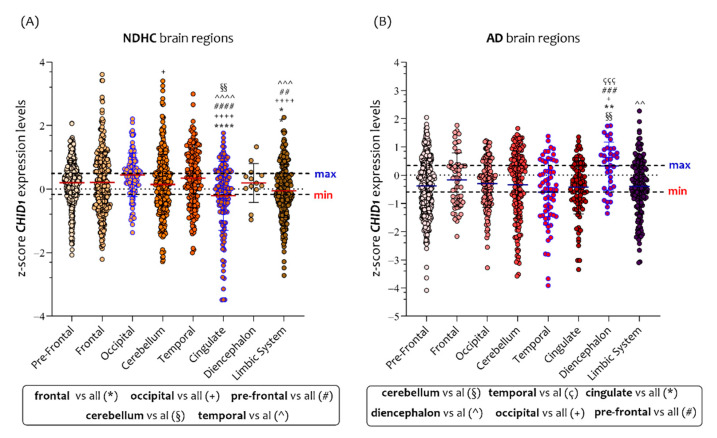
*CHID1* levels are differentially expressed in brain regions of NDHC subjects and AD patients. Analyzing the NDHC individual brain regions, we observed that the highest *CHID1* expression levels were in the occipital cortex, while the lowest in the region of cingulate (**A**). As regards the AD patients’ brain regions, we observed that the highest *CHID1* expression levels were in the diencephalon, while the lowest at the region of the temporal cortex (**B**). See the text for details. Data in the figure are indicated as z-score intensity expression levels and presented graphically as violin dot plots. *p* values < 0.05 were considered to be statistically significant (ns = not significant; +, * *p* < 0.01; §§, ##, ^^, **, *p* < 0.001; ^^^, ççç, ###, *p* < 0.0001; ++++, ####, ^^^^, ****, *p* < 0.00001).

In analyzing the NDHC subjects’ eight brain regions, we observed that the highest *CHID1* expression levels were in the occipital cortex, while the lowest were in the cingulate cortex (Figure 6A). As regards the AD patients’ eight brain regions, we observed that the highest *CHID1* expression levels were in the diencephalon, while the lowest were in the temporal cortex (Figure 6B). Significant differences in *CHID1* expression levels were found by comparing the frontal cortex (*p* < 0.0001, *p* = 0.001), prefrontal (*p* < 0.0001, *p* = 0.001), occipital (*p* < 0.0001, *p* < 0.0001), (*p* = 0.001), and temporal (*p* < 0.0001, *p* < 0.001) brain regions with the cingulate and limbic system in NDHC subjects (Figure 6A). Furthermore, we observed that *CHID1* expression in the cerebellum was significantly lower than in the occipital lobe (*p* < 0.01) (Figure 6A). 

As regards the AD patients’ brain regions, we observed significant differences in *CHID1* expression levels by comparing the cerebellum (*p* = 0.001), temporal (*p* < 0.0001), cingulate (*p* = 0.001), occipital (*p* = 0.01), and prefrontal (*p* < 0.0001) brain regions with the diencephalon (Figure 6B). Furthermore, we observed that *CHID1* expression in the limbic system was significantly lower than in the diencephalon (*p* < 0.001) (Figure 6B). 

In analyzing the *CHID1* expression levels in the eight brain regions in NDHC subjects and AD patients, we highlighted a significant difference only in NDHC subjects’ limbic system region, in which males had significantly lower levels than females (*p* < 0.0001) (Figure S1A). No significant difference between the sexes was observed in the different brain regions of AD patients (Figure S1B).

**Figure 7:**
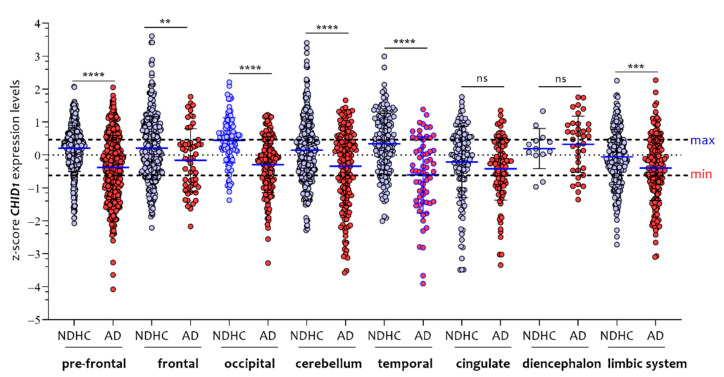
*CHID1* expression levels comparative analysis in eight brain regions of NDHC subjects and AD patients. By analyzing the individual brain regions according to the disease, we observed that the *CHID1* expression levels were significantly higher in NDHC than in AD in all brain regions, with the exception of the diencephalon and cingulate regions. Data in the figure are indicated as z-score intensity expression levels and presented graphically as violin dot plots. *p* values < 0.05 were considered to be statistically significant (ns = not significant; ** *p* < 0.001; *** *p* < 0.0001; **** *p* < 0.00001).

By analyzing the individual brain regions according to the disease, we observed that the *CHID1* expression levels were significantly higher in NDHC than in AD in all brain regions, with the exception of the diencephalon and cingulate regions (Figure 7).

### 3.4. ROC Analysis Confirmed the Diagnostic Ability of CHID1 to Discriminate AD Patients from NDHC Subjects

**Figure 8:**
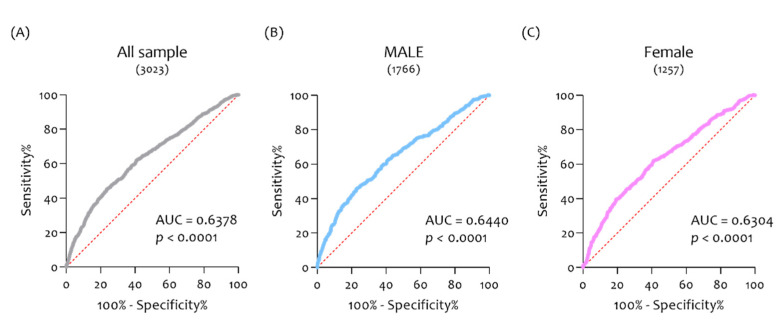
*CHID1* brain expression levels as a prognostic marker of AD. In order to evaluate the potential diagnostic ability of *CHID1* to discriminate against the AD patients from NDHC subjects, we performed a receiver operating characteristic (ROC) analysis. *CHID1* expressed fair diagnostic ability (AUC = 0.6368, *p* < 0.0001) (**A**). This ability was maintained for both males (AUC = 0.6440, *p* < 0.0001) (**B**) and females (AUC = 0.6304, *p* < 0.0001) (**C**).

In order to evaluate the potential diagnostic ability of *CHID1* to discriminate the AD patients from NDHC subjects, we performed a receiver operating characteristic (ROC) analysis. We showed that *CHID1* express a fair diagnostic ability to discriminate the AD from NDHC (AUC = 0.6368, *p* < 0.0001) (Figure 8A). This ability was maintained for both males (AUC = 0.6440, *p* < 0.0001) (Figure 8B) and females (AUC = 0.6304, *p* < 0.0001) (Figure 8C).

## 4. Discussion

Here, we have shown that the gene expression of *CHID1* was downregulated in the brain of AD patients compared to NDHC subjects, and both in NDHC and in AD patients, the expression levels were strongly correlated with *NRGN* and *CALB1*. Only in AD patients, the *CHID1* expression levels were positively correlated with *IBA1* levels. A closer investigation of the *CHID1* expression levels in the different brain regions showed significant correlation differences between NDHCS and AD in prefrontal, frontal, occipital, cerebellum, temporal, and limbic system. Sex-related differences in *CHID1* expression were only highlighted in NDHC subjects’ limbic system region in which males had significantly lower levels than females.

In recent years, the use of datasets available in public databases has grown exponentially. Several research groups, including ours, have extensively used the analysis of public transcriptome datasets for the identification of novel pathogenic pathways and therapeutic targets in several human pathologies [25,26,89,90,91,92,93,94] including neurodegenerative disease [6,12,13,25,95,96] and cancer [14,97,98,99]. Through a meta-analysis of public array datasets, it is possible to increase the statistical power to obtain a more precise estimate of gene expression differentials, and assess the heterogeneity of the overall estimate. Meta-analysis is relatively inexpensive since it makes comprehensive use of already available data and represents a vast source of information that could make a difference in setting up highly targeted experimental strategies.

To date, information regarding the potential role played by CHID1 in CNS has remained very poor. On the contrary, great attention has been paid to other chitinase-like proteins such as CHI3L1, CHI3L2, CHIA, and CHIT1 and their role in various inflammatory processes [100,101]. In 2016, our group demonstrated, for the first time, that the *CHID1* expression levels were significantly downregulated in the brains of LOAD patients and were not related to sex [13]. In this manuscript, we have shown that *CHID1* expression levels were significantly downregulated in AD patients and correlated positively with *NRGN* and *CALB1* and negatively with *IBA1* and CD31 in the brain of NDHC subjects. 

Furthermore, in the 1266 brain sample biopsies of males and in the 587 of female NDHC subjects, the *CHID1* expression levels were inversely correlated with age. These results are in line with the data previously observed by our group in a small cohort of samples [13]. In the past, we have speculated that CHI3L1 could play a role in the cytoskeletal structure [11,16]. Such data have also been partially verified for *CHID1* [16]. Indeed, it has been shown that CHID1 suppressed macrophage cytoskeletal rearrangements in response to CCL2 [102]. Furthermore, using the online tool “The Human Protein Atlas”, we have observed that CHID1 protein was localized in the nucleoplasm and in intermediate filaments. This potential interaction with the cytoskeleton could partially explain the inverse correlation with age as well as the reduction in the brain in AD subjects. Numerous studies analyzing human postmortem tissue, animal models, and cellular paradigms indicate that AD pathology has a deleterious effect on the pathways governing actin cytoskeleton stability [103]. These conditions have also been demonstrated during brain aging [104]. It is known that the cytoskeleton is an abundant and broadly expressed structure that plays critical functions in many cellular processes ranging from cell motility to controlling cell shape and polarity. In light of this, cytoskeleton function in neurons is crucial for the morphological changes that occur in the brain throughout life. We could hypothesize that the CHID1 expression is linked to the cytoskeleton and that it can play a role at the level of the dendritic spines. The cytoskeleton alteration mediated by CHID1 could be one of the key events contributing to the initial phases of aging in the brain or during the AD progression. Furthermore, we showed that both in males and females during aging, in healthy subjects and in AD patients, the expression levels of *CHID1* and *NRGN* were closely related. There is a close relationship between NRGN and rearrangement of the cytoskeletal structures of neurons [105]. It is known that NRGN is a postsynaptic protein primarily expressed in the brain, particularly in dendritic spines. Because of its abundant and preferential neuronal expression, NRGN has been identified as a potential marker of age-related neurodegeneration. Synaptic loss is an early pathologic substrate of AD. Actually, the CSF protein levels are used as AD progression markers [106]. The strong correlation identified between *CHID1* and *NRGN* brain expression levels allows us to hypothesize a probable common role between the two genes.

An extremely interesting result highlighted during our investigation was the strong correlation between *CHID1* and *CALB1* expression levels in the brain of NDHC subjects and AD patients. *CALB1* is one of the major calcium-binding proteins that plays a critical role in preventing neuronal death as well as maintaining calcium homeostasis [41]. Decreased *CALB1* expression levels represent an index of neuronal death. It has been shown that CALB1 removal from amyloid precursor protein/presenilin transgenic mice aggravates AD pathogenesis, suggesting that CALB1 has a critical role in AD pathogenesis [41]. The fact that the *CHID1* and *CALB1* expression levels are strongly correlated suggests not only that *CHID1* is localized at the level of neurons but that it can play a role in structural maintenance. Other chitinases have already shown multifaceted functions according to the cells in which they are expressed. CHID1 could also perform different functions depending on the cellular environment in which it is expressed. Not surprisingly, in our analysis, we have observed a positive correlation between the expression levels of *CHID1* and *IBA1* only and exclusively at the level of the brains of AD patients, regardless of gender. This would suggest that CHID1 could be also associated with microglial activation. The association between CHID1 and macrophage lineage activation represents one of the few pieces of information currently available on the role played by this gene [25].

*CHID1* brain expression levels were different in the eight brain regions analyzed. In NDHC subjects, the highest levels were in the occipital lobe, a region involved in functions linked to visual perception, while the lowest were in the cingulate cortex, a region involved in certain higher-level functions such as attention allocation, decision-making, morality, impulse control, and emotion. In the brains of AD patients, this trend was completely distorted, finding the lowest levels in the temporal region involved in processing sensory input visual memory and comprehension of language, and the highest levels in the diencephalon, which includes several regions with functions associated with recognition of the sensory impulses of heat, cold, pain, pressure, control of eye movement, and hearing responses.

Sex differences in *CHID1* expression levels were observed only in the limbic system region in NDHC subjects, in which males had significantly lower levels than females. These results would suggest that *CHID1* could play different roles in healthy subjects compared to AD patients, and that its function could be potentially related to the cognitive functions of the brain regions to in which it is involved.

Summing up, our analysis shows that *CHID1* has an opposite trend in the CNS compared to other CLPs. Indeed, while the levels of CHI3L1 and CHI3L2 in AD patients tend to increase leading to hypothesis of a potential immunological role, *CHID1* levels tend to decrease both with age and in neurological diseases such as AD. This reduction associated with both physiological and pathological aging as in AD could suggest a structural, rather than immunological, role in the neurons.

## 5. Conclusions

The biological function of CHID1 remains poorly explored and still unknown. Our manuscript has tried to partially shed light on the potential involvement of its expression in AD disease. Previously, our group identified three other chitinases involved in AD [13,107,108]. While the CHIT1, CHI3L1, and CHI3L2 expression levels tend to increase in the brain of AD patients, most likely because they are linked to neuroinflammation and innate immune cell activation, in the opposite manner, the CHID1 levels tend to decrease, and we hypothesized this is because they are linked to neuron death. A therapeutic strategy that significantly reduces the neuroinflammation in the brain of AD patients could consequently be reflected as a reduction in the expression levels of CHIT1, CHI3L1, and CHI3L2. Regarding the *CHID1* expression levels, therapy capable of reducing or slowing neuronal death could be verified by an increase in *CHID1* expression levels. Most likely, the action of CHID1 inside neurons is attributable to structural, cytoskeletal functions, but further studies are needed to prove this hypothesis. We recently hypothesized that the role of CHID1 can be also involve the nuclear level [16]. 

In light of this information, the sexual differences in the CHI3L1 and CHI3L2 expression levels, highlighted in e recent papers [13] and also partially found for CHID1, could open the door to targeted strategies involving gender-specific therapies. Unfortunately, as for the differential diagnosis, we cannot answer exhaustively if CHID1 is a unique marker of AD disease, but it represents an excellent starting point for further investigations in order to investigate the potential role of *CHID1* in the diagnosis and progression of this disease.

## Figures and Tables

**Table 1:** Datasets selected.

N°	Dataset	Organism	Platform		NDHC	AD	References
1	GSE33000	*Homo sapiens*	GPL4372	HBTRC	144	310	[51]
2	GSE28894	*Homo sapiens*	GPL6104	NIA	29	0	[52]
3	GSE35978	*Homo sapiens*	GPL6244	SMRI	13	0	[53]
4	GSE15745	*Homo sapiens*	GPL6104	UMBB	132	0	[54]
5	GSE44772	*Homo sapiens*	GPL4372	HBTRC	273	387	[55]
6	GSE36192	*Homo sapiens*	GPL6947	NIA	379	0	[56]
7	GSE60862	*Homo sapiens*	GPL5175	UKBEC	226	0	[57]
8	GSE118553	*Homo sapiens*	GPL10558	MRC-LBB	72	167	[58]
9	GSE25219	*Homo sapiens*	GPL5175	BTBDDUM	27	0	[59]
10	GSE71620	*Homo sapiens*	GPL11532	PBTDP	208	0	[60]
11	GSE5392	*Homo sapiens*	GPL96	SMRIC	3	0	[61]
12	GSE36980	*Homo sapiens*	GPL6244	KU	47	32	[62]
13	GSE26927	*Homo sapiens*	GPL6255	BNEN	4	11	[63]
14	GSE84422	*Homo sapiens*	GPL570	MSBB	28	74	[64]
15	GSE5281	*Homo sapiens*	GPL570	ADCs	74	87	[65]
16	GSE48350	*Homo sapiens*	GPL570	ADC	93	80	[66]
17	GSE11882	*Homo sapiens*	GPL570	MSBB	93	0	[67]
18	GSE28146	*Homo sapiens*	GPL570	BBADRCUK	8	22	[68]

**Table 2:** Sample stratification.

	Age Stage	NDHCS	AD
1	53–65 middle-age	734 = 582 male + 152 female	59 = 34 male + 25 female
2	65–75 senior	425 = 291 male + 134 female	219 = 75 male + 144 female
3	76–89 elderly	493 = 328 male + 165 female	639 = 361 male + 278 female
4	90–99 nonagenarian	179 = 61 male + 118 female	235 = 187 male + 48 female
5	>100 centenarian	22 = 4 male + 18 female	18 = 13 male + 5 female
	Total sample	1853	1170

NDHC = non-demented healthy controls subjects; AD = Alzheimer’s disease patients.

**Table 3:** Brain regions analyzed.

N°	Brain Regions	Abbreviations	Brain Portions	n° of Sample	Age
1	prefrontal	PFC	prefrontal cortex; dorsolateral prefrontal cortex; dorsolateral prefrontal cortex; medial prefrontal cortex; ofc (orbitofrontal cortex); orbitofrontal cortex; orbital prefrontal cortex; ventral forebrain; ventrolateral cortex; ventrolateral prefrontal cortex	1045 = 784 male + 261 female	51.18 ± 21
2	frontal	FC	frontal cortex frontal pole (Brodmann area 9, 10); medial frontal cortex	868 = 577 male + 291 female	51.07 ± 26.08
3	occipital	OC	occipital cortex; primary visual cortex; visual cortex	286 = 207 male + 79 female	55.16 ± 25.44
4	cerebellum	CB	cerebellar cortex; cerebellum; upper (rostral) rhombic lip	1009 = 693 male + 316 female	50.9 ± 24.52
5	temporal	TP	inferior temporal cortex; primary auditory cortex; superior temporal cortex; superior temporal cortex (Brodmann area 22); temporal cortex; ventral head of the caudate nucleus	466 = 174 male + 292 female	47.77 ± 29.01
6	cingulate	CYN	anterior cingulate; caudal ganglionic eminence; lateral ganglionic eminence; medial ganglionic eminence; medial temporal gyrus; postcentral gyrus; posterior cingulate; posterior cingulate cortex; subpial grey matter lesions from the frontal gyri; superior frontal gyrus	319 = 196 male + 123 female	61.35 ± 22.55
7	diencephalon	DIE	basal ganglia; dorsal thalamus; putamen; striatum; nucleus accumbens; substantia nigra; thalamus; mediodorsal nucleus of the thalamus	450 = 304 male + 146 female	55.41 ± 24.47
8	limbic system	LS	amygdala; entorhinal cortex; hippocampus	563 = 356 male + 207 female	56.83 ± 26.31

## Data Availability

The datasets analyzed during the current study are available in GeoDataset. See Table 1 for the reference.

## Supplementary Materials

The following are available online at https://www.mdpi.com/article/10.3390/cells10040882/s1, Figure S1: Significant difference in NDHC subjects’ limbic system region.
