# Supplementary material for: Brain CHID1 Expression Correlates with NRGN and CALB1 in Healthy Subjects and AD Patients
Source: Cells. 2021 Apr 13;10(4):882. doi: 10.3390/cells10040882 (PMC8069241; doi:10.3390/cells10040882)
Supplement: Supplementary file 1 [file cells-10-00882-s001.pdf]

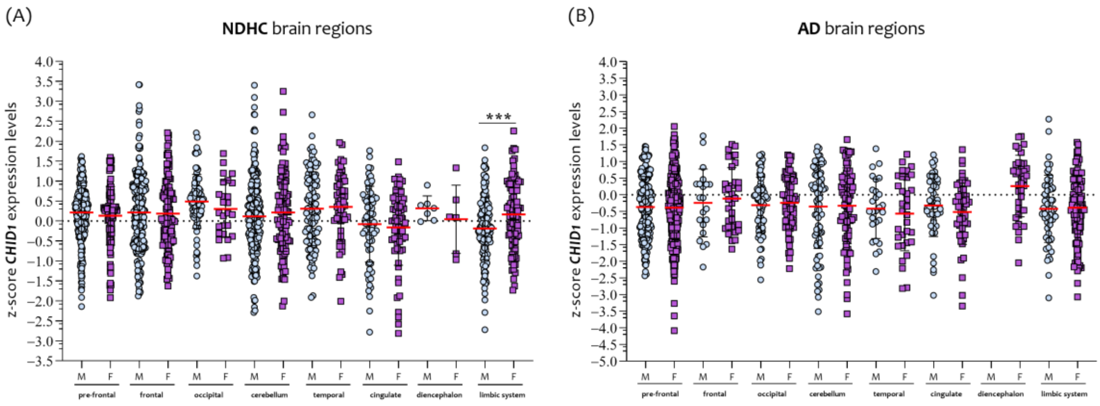

Figure S1. Significant difference in NDHC subjects' limbic system region (males had significantly lower levels than females, $p < 0.0001$) (Figure S1A). No significant difference between the sexes was observed in the different brain regions of AD patients (Figure S1B).
